# Temperature Effects on the Cryphonectria hypovirus 1 Accumulation and Recovery within Its Fungal Host, the Chestnut Blight Pathogen *Cryphonectria parasitica*

**DOI:** 10.3390/v15061260

**Published:** 2023-05-27

**Authors:** Pedro Romon-Ochoa, Olivia Smith, Alex Lewis, Quirin Kupper, Wajeeha Shamsi, Daniel Rigling, Ana Pérez-Sierra, Lisa Ward

**Affiliations:** 1 Forest Research, Plant Pathology Department, Alice Holt Research Station, Surrey GU104LH, UK; olivia.smith@forestresearch.gov.uk (O.S.); lisa.ward@forestresearch.gov.uk (L.W.); 2 Forest Research, Tree Health Diagnostics and Advisory Service (THDAS), Alice Holt, Surrey GU104LH, UK; alex.lewis@forestresearch.gov.uk (A.L.); ana.perez-sierra@forestresearch.gov.uk (A.P.-S.); 3 Swiss Federal Institute for Forest, Snow and Landscape Research WSL, Zuercherstrasse 111, 8903 Birmensdorf, Switzerland; quirin.kupper@wsl.ch (Q.K.); wajeeha.shamsi@wsl.ch (W.S.); daniel.rigling@wsl.ch (D.R.)

**Keywords:** Cryphonectria hypovirus 1, fungal growth, hypovirulence, temperature, accumulation, recovery

## Abstract

Biological control of *Cryphonectria parasitica* fungus, the causal agent of chestnut blight, by virus infection (hypovirulence) is an effective control strategy against chestnut blight in Europe and some parts of North America. The most studied mycovirus is the Cryphonectria hypovirus 1 (CHV1) type species of the *Hypoviridae* family. In this study, the CHV1 virus was studied within some highly infected British isolates of *Cryphonectria parasitica*, gained in the past through co-culture transmissions. The effects of six temperatures (5–30 °C, in 5 °C steps) on six infected isolates (three with viral strain E-5, and other three with viral strain L-18) and their respective negative non-infected controls, three isogenic virulent fungal isolates, were examined. Experiments were performed with the nine isolate types with three replicates on potato dextrose agar (PDA) with cellophane sheets per isolate and temperature. A recently developed rapid, specific, quantitative reverse transcription PCR (RT-qPCR) screening method was used. This enabled quantifying the concentration (nanograms per microliter or copy numbers) of the virus within each isolate repetition. The presence of the virus had a significant negative effect between 20 and 25 °C on the *C. parasitica* growth rate, which was anyway highly influenced by and positively correlated with the temperature. The temperature clearly determined the virus accumulation and its recovery from cold or heat, and the virus optimum temperature was estimated at 15–25 °C.

## 1. Introduction

Viruses occur commonly in all major groups of true fungi [1,2] and some Oomycetes [3,4]. Fungal viruses are classified into nine double-stranded RNA families (*Chrysoviridae*, *Spinoreoviridae, Partitiviridae*, *Totiviridae*, *Polymycoviridae*, *Quadriviridae, Megabirnaviridae*, *Amalgaviridae*, and *Curvulaviridae*) and the genus *Botybirnavirus*, twelve single-stranded RNA families (*Endornaviridae, Barnaviridae*, *Hypoviridae*, *Narnaviridae*, *Alphaflexiviridae*, *Gammaflexiviridae*, *Hadakaviridae, Botourmiaviridae, Mitoviridae, Mymonaviridae, Yadokariviridae*, *and Phenuiviridae*), two reverse-transcribing virus families *Metaviridae* and *Pseudoviridae*, and one ssDNA family *Genomoviridae* [5]. Some act as antagonists of their fungal hosts, decreasing their growth, pigmentation, conidiation and/or virulence. Examples include the fungal genera *Cryphonectria* [6], *Diaporthe* [7], *Fusarium* [8], *Rosellinia* [9], etc. Some other fungal viruses develop neutral effects on their host, e.g., Hymenoscyphus fraxineus mitovirus 1 [10], or have a positive association with their host such as Nectria radicicola virus L1 [11].

*Cryphonectria parasitica*, a fungus with an Asiatic origin (native in China, Japan, and Korea) [12], causes chestnut tree blight in North America [13], Europe [14], and England [15] in geographical areas where the pathogen has been introduced multiple times, mainly by trade in chestnut plants and reforestation.

England was considered free of chestnut blight until 2011, when *C. parasitica* infections were discovered on 90 young saplings of sweet chestnut planted in a nursery farm in Warwickshire. They originated from the same nursery in Europe; the saplings were imported and planted in 2007 and some trees died and were replaced in 2010. This stimulated surveys between 2011 and 2012 where the fungus was identified on recently planted saplings at a further eight orchard sites located in Devon, Herefordshire, Kent, Norfolk, Somerset, and Sussex. All affected trees were eliminated. In 2013, United Kingdom introduced tighter import controls, meaning that the movement of sweet chestnut trees in, around and out of England needed to be accompanied by official documentation confirming that they were from an area free of the disease. In August 2016, chestnut blight was confirmed on a recently planted tree in Kent that was removed. Until 2016, all findings of the disease in the UK were exclusively in orchards or recently planted individual young trees, and therefore their eradication was relatively easy. However, in December 2016, *C. parasitica* was isolated from four mature trees growing in a car park in Devon. Additional Forestry Commission of England and Animal and Plant Health Agency surveys were initiated, and *C. parasitica* was subsequently diagnosed in a woodland about 1 km away from the last infected site. A trace-forward and trace-back exercise was initiated, which revealed multiple positive findings in England between 2017 and 2020.

Eradication efforts, cutting and burning the infected trees/branches, are especially difficult in some areas and have mostly failed [16]. Therefore, interest in biocontrol is growing. The fungus can be infected, for example, by a mycovirus called Cryphonectria hypovirus 1 (CHV1), which is the prototype of the family Hypoviridae [17]. Hypoviruses are RNA viruses located in the cytoplasm membrane vesicles of their fungal hosts. They are without a coat protein, and their replicative form is dsRNA [18]. CHV1 acts as a biocontrol agent of chestnut blight in Europe and some parts of North America (Virginia, Wisconsin, Maryland), where it has been released, because it induces reduced growth, pigmentation, sporulation, and virulence on its fungal host [16]. This virus is vertically transmitted (to the progeny) through fungal conidia–with rates between 77 and 100% depending on fungal and viral types [19]. It can also be horizontally transmitted through anastomosis between mycelia of two isolates of the same vegetative compatibility group (VCG) at around 100% transmission–or between different VCGs at 0–100% transmission [20,21]. In England, CHV1 was detected for the first time in November 2017 [15]. It has been recorded in low frequency and concentration.

There is a low prevalence and load thus of CHV1 in the Forest Research collection of English isolates. The low temperatures in England, with CHV1’s more common occurrence at higher loads in the temperate areas of continental Europe, led to a hypothesis that the concentration of this mycovirus might be influenced by climate, particularly temperature.

Recently highly CHV1-infected isolates of VCG EU-10 (dominant in London and ranging between 234.4 and 407.1 ng/µL of viral amplicon after RT-PCR) and VCG EU-9 (dominant in Devon and ranging between 343.0 and 612.8 ng/µL of viral amplicon after RT-PCR) were achieved by co-culture transmissions with CHV1-infected cultures from Switzerland [22]. The aims of the present study were: (i) to analyse the influence of six temperatures (5–30 °C, in 5 °C steps) on the mycovirus accumulation levels in those highly infected British isolates of *C. parasitica*; (ii) to assess the relationship between temperature, mycovirus accumulation, and *C. parasitica* growth; and (iii) to determine the capacity of the virus to recover after each temperature treatment.

## 2. Material and Methods

### 2.1. Viral and Fungal Strains

Viral and fungal strains used in the current study are shown in Table 1. Two CHV1 strains were tested: CHV1-M2273 haplotype E-5, and CHV1-M2357 haplotype L-18, previously horizontally transmitted to the British fungal isolates FTC687, WAP706, and POWP709 by coculture of virus-infected donor and recipient fungal isolates on a PDA plate (9 cm in diameter) as described previously [23].

On the other hand, three non-infected virulent British fungal isolates were used: FTC687 (isolated in 2021 from London), WAP706, and POWP709 (both isolated in 2021 from Devon) [24], belonging to the EU-10, and EU-9 VC groups respectively.

### 2.2. Culture Conditions

The effect of temperature on the mycelial growth was investigated by incubating the cultures in the dark in six independent MIR-554-PE growth chambers (PHCBI, Loughborough, UK) at 5, 10, 15, 20, 25, and 30 °C. Each isolate had three replicates; consequently 162 dishes were measured after one week. Two perpendicular lines were drawn on the back side of each dish of 20 mL of potato dextrose agar (PDA) medium overlaid with a cellophane sheet. A mycelial plug (5 mm in diam.) from each 7 days-old isolate was placed on the intersection of both lines on top of a squared cellophane sheet. The response variables were the two diameters measured along the lines. After the experimental week, RNA was extracted, and the virus was quantified as described below.

### 2.3. RNA Extraction and Reverse Transcription PCR

Fungi were cultivated on PDA plates covered by cellophane membranes and incubated at the different temperatures. After one week, the diameters of the cultures were traced and measured. The mycelium of each culture was removed from the cellophane and weighed. RNA was extracted with the RNeasy Plant Mini Kit (Qiagen, Manchester, UK), with an on-column simultaneous DNA digestion with the following modifications (per tube). RLT lysis buffer (450 µL) prepared with 6 ng/µL glycogen as RNA carrier (Sigma-Aldrich, Gillingham, UK) was added to the tissue. Each sample was homogenized using a QIAshredder column. Final eluates were stored at −80 °C. RNA was quantified using a Nanodrop spectrophotometer (Fisherscientific, Loughborough, UK).

RNA was then reverse transcribed and amplified through a one-step quantitative reverse transcription polymerase chain reaction (RT-qPCR), using One Step PrimeScript III RT-qPCR kit (TaKaRa Bio, Saint-Germain-en-Laye, France). Real-time PCRs were carried out on a LightCycler 480 (Roche, Welwyn, UK), targeting both CHV1 and the fungal actin mRNA. For the assay, 400 nM of each of the primers CHV1-F (5′-TGAGGAACGTCAACTTCG-3′), CHV1-R (5′-TTGTGACGACGGAAATAATC-3′), CpActinmRNAF1 (5′-CCATGGTATCATGATTGGTATG-3′), and CpActinmRNAR1 (5′-TACCGCAGAGTCAGGATA-3′) were used, and 400 nM of each of the probes HVEP1 Fluo (5′-56-FAM/TGACACGGAAGCTGAGTGTC/3BHQ1/-3′) and CpActinmRNAP1 (5′-56-JOE/TCATCACCAACATACGAGTCCTTCTG/3BHQ1/-3′).

Twenty microliters reaction volume was used, comprising per reaction 10 µL 2X One Step Primescript III RT-qPCR Mix, 0.4 µL of each primer and probe, 6.6 µL RNase free water, and 1 µL of RNA sample. Each sample was assayed in triplicate. Thermal cycling conditions were 50 °C for 30 min and 95 °C for 50 s, followed by 40 cycles of 95 °C for 25 s and 53 °C for 1 min. Fluorescent detection occurred at the end of each 53 °C step. The cycle threshold (Ct) value was calculated automatically using the LightCycler software with absolute quantification using second derivative maximum setting with 465–510 and 533–580 nm channel filters respectively for analysing the specific amplicons and the actin RNA quality control [22].

**Figure 1:**
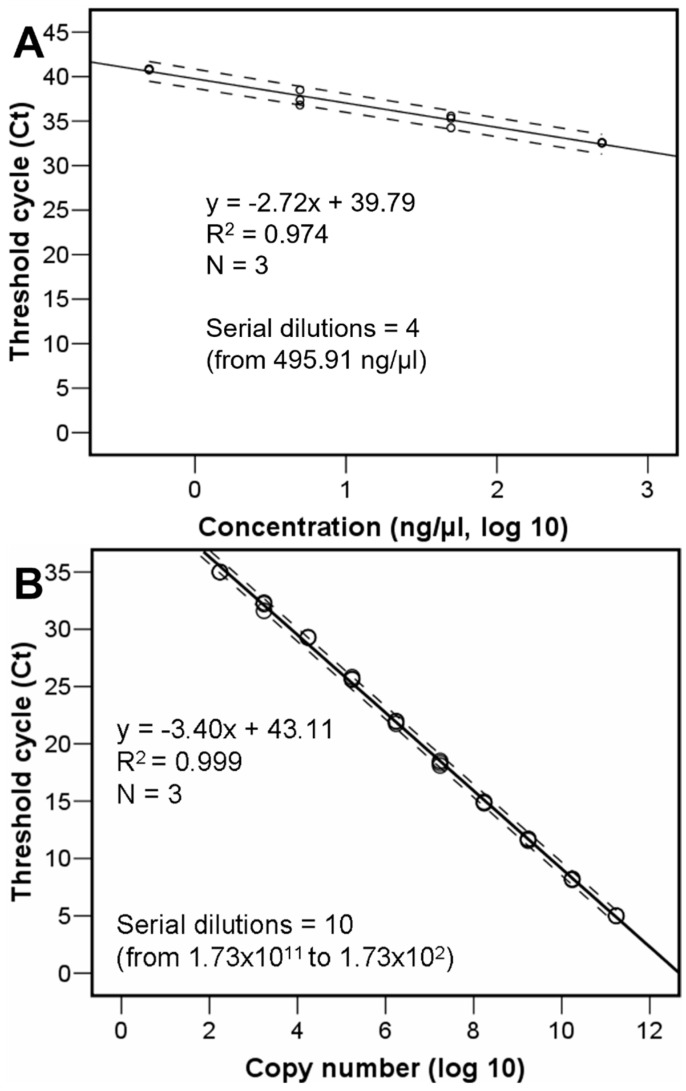
Regression equations respectively relating the CHV1 nanograms (**A**) and copy numbers (**B**) per microliter with threshold cycle values.

The 94 bp qPCR product was synthesised de novo and cloned into a plasmid pUC-GW-Kan (Azenta Life Sciences, Leipzig, Germany). The fragment copy number, actual number of CHV1 virus particles, was estimated using a ten point 1:10 serial dilution of the plasmid and subsequent posterior regression equation analyses (Figure 1B). For calculating the copy number in the original plasmid aliquot, the copy number was calculated using the following formula: number of copies/µL = [(6 × 10^23^) × (DNA concentration, 500 ng/µL)/molecular weight of one plasmid], where Avogadro’s number is the number of copies per mole, DNA concentration is given in grams per microliter, and the molecular weight of one plasmid is in grams per mole, assuming a plasmid size of 2627 bp and a 1 bp molecular weight of 660 g/mole. The threshold cycle values permitted also calculating the viral concentration (in nanograms per microliter) after building up an equation with a four points serial dilution of known concentrations standard (Figure 1A) submitted to the same RT-qPCR.

The complete experimental protocol but without cellophane was repeated to test for potential recoveries from cold or heat. After the temperature treatments, a 5 mm diameter mycelial plug per repetition was further incubated in 1 mL malt extract broth (MEB) in 2-mL Eppendorf tubes at 25 °C for one week, before quantifying the virus concentration and copy number as above.

### 2.4. Statistical Analyses

Differences in virus load were analysed in two ways–using viral concentration in nanograms per microliter and by using the variable virus copy number per microliter. Multivariate general linear model ANOVA-type-III analyses were used to assess for significant differences, using Tukey’s HSD adjustments for determining statistical significance with IBM SPSS 13.0 software, and building up regression equations when appropriate.

## 3. Results

**Figure 2:**
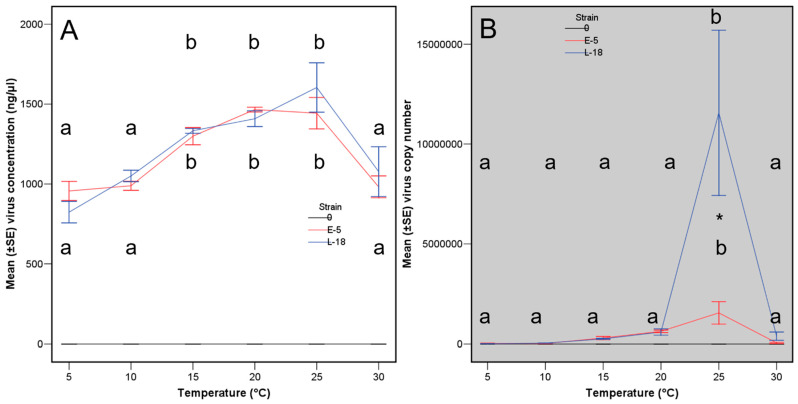
Virus concentration (**A**) and virus copy number (**B**) at each temperature for viral strains E-5 (red lanes) and L-18 (blue lanes). Lanes with the same letter are not significantly different based on a Tukey′ s test. Lanes with an asterisk indicate significant differences between the two virus haplotypes.

Virus concentration, within *C. parasitica* colonies along the trial with cellophane, was influenced by the original virus concentration in the inoculated plugs (Fisher variances ratio, F = 4.51, Probability, *p* = 0.002), and the temperature, but was independent of the haplotype of the two tested virus strains (Table 2 top analysis, Figure 2A). The maximum virus concentration was achieved at 15 to 25 °C. Viral concentration (in nanograms per microliter) marked the differences better than using virus copy number per microliter in all assays using cellophane because the copy number data was highly influenced by the much higher values at 25 °C of the viral strain L-18 (Table 2, Figure 2B). Thus, virus concentration resulted more reliable data.

**Figure 3:**
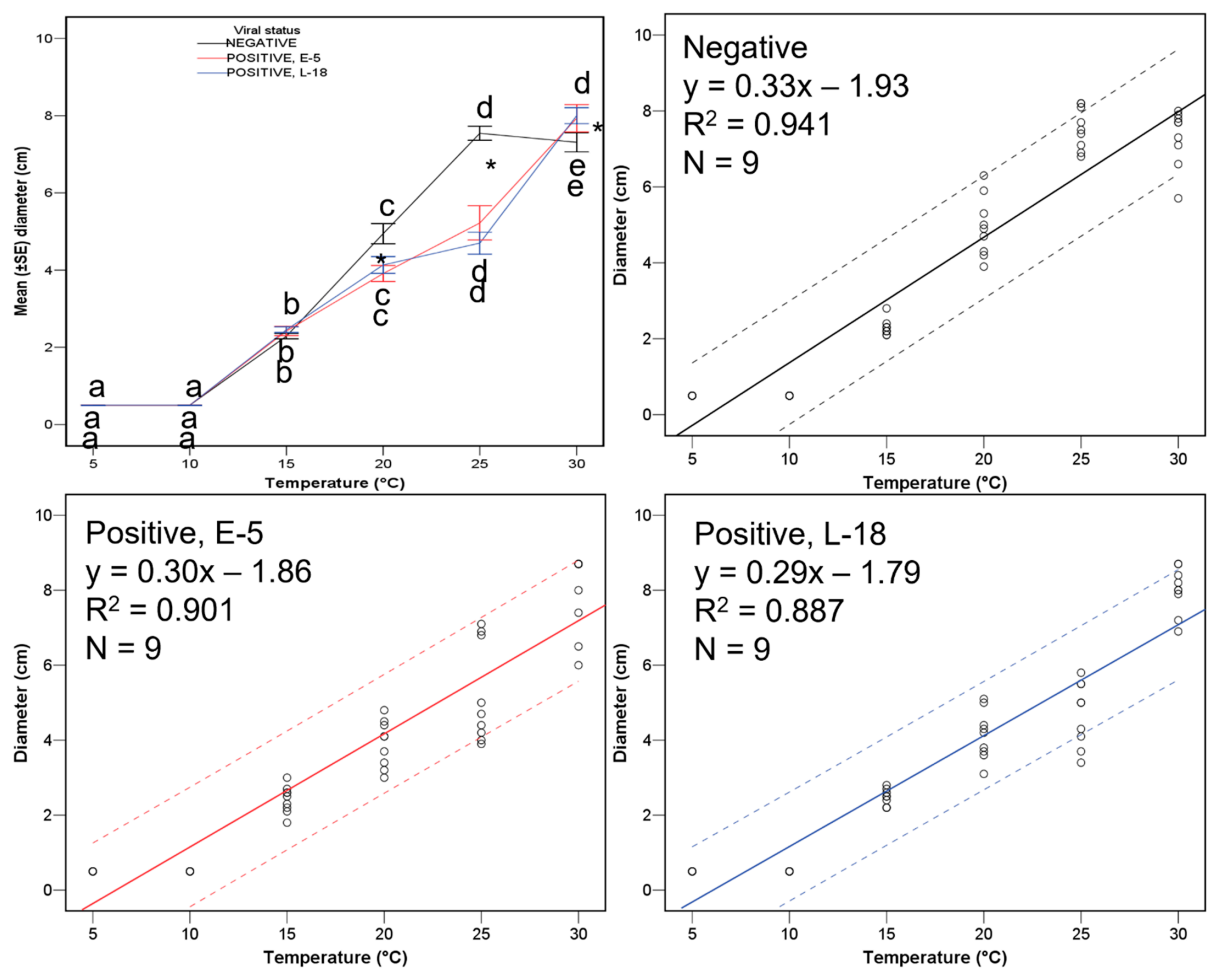
Maximum diameter (cm) at each temperature for viral negative and positive isolates, with representation of the potential regression equations relating growth and temperature for each viral strain (nil, black lanes; E-5, red lanes; L-18, blue lanes). Lanes with the same letter are not significantly different based on a Tukey′ s test. Lanes with an asterisk indicate significant differences between the two virus haplotypes or virulent isogenic isolates.

The colony diameter was positively influenced by increasing temperatures, and negatively influenced due to the virus presence between 20 and 25 °C, with the virus-infected cultures slightly smaller than the non-infected ones at 20 °C and approximately half the size at 25 °C (Table 3, Figure 3). There were no significant differences between the two viral strains. Therefore, CHV1 presence did significantly impair the growth of *C. parasitica* isolates between the mentioned two temperatures. In general, the temperature significantly affected the fungal growth rate. At a temperature of 30 °C, *C. parasitica* grew the most and at 5/10 °C the least. These data (high square R values) supported building up respective regression equations relating the fungal growth diameter and the temperature to the viral negative and positive isolate repetitions (Figure 3).

**Figure 4:**
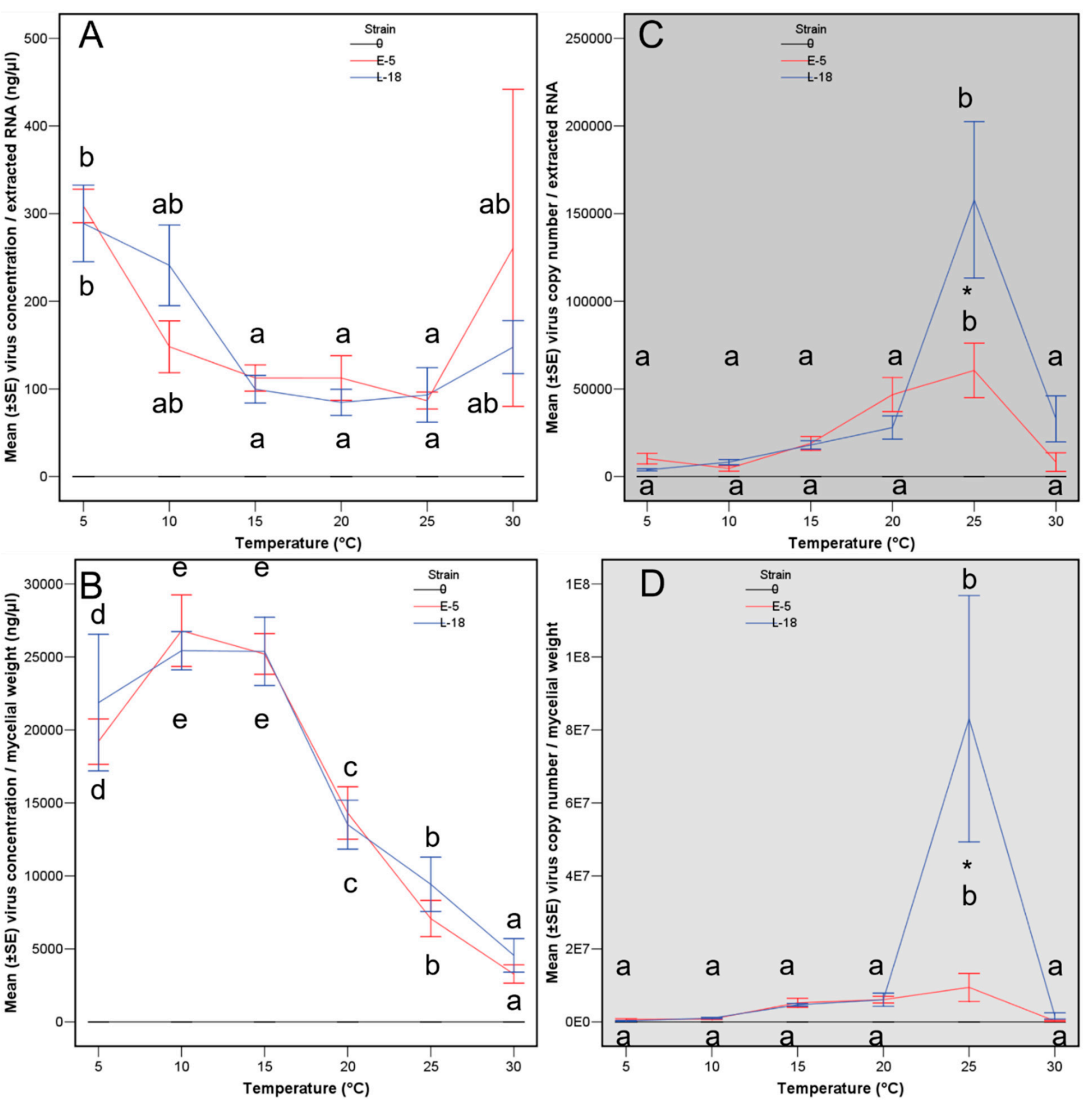
Viral concentration at each temperature and viral strain divided by extracted RNA (**A**) or mycelium weight (**B**) plus viral copy number at each temperature and viral strain divided per extracted RNA (**C**) or mycelium weight (**D**). Viral strains (E-5, red lanes; L-18, blue lanes). Lanes with the same letter are not significantly different based on a Tukey′ s test. Lanes with an asterisk indicate significant differences between the two virus haplotypes.

If we divide the virus concentration by the extracted RNA concentration, the virus concentration was significantly dependent on temperature, resembling a negative Gauss curve (Figure 4A), as opposite to the parabola in Figure 2A. The same parameter divided by the weighed mycelial tissue was influenced by the temperature and the original virus concentration (F = 7.545, *p* = 0.0001) (Table 2 most bottom analysis, Figure 4B).

**Figure 5:**
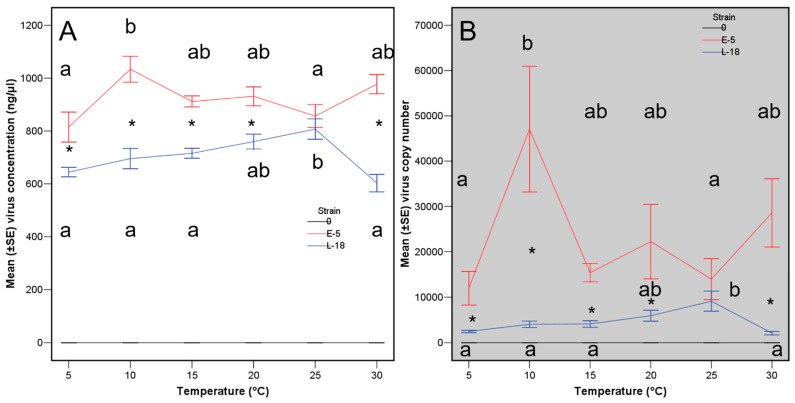
Virus concentration (**A**) and virus copy number (**B**) at each temperature for viral strains E-5 (red lanes) and L-18 (blue lanes) after additional incubation in broth at 25 °C for one week. Lanes with the same letter are not significantly different based on a Tukey′ s test. Lanes with an asterisk indicate significant differences between the two virus haplotypes.

The capacity of the viral strain E-5 to recover in concentration and copy numbers was always greater than that of strain L-18 (Table 4, Figure 5A,B), at all the tested original temperatures except from 25 °C.

## 4. Discussion

Cryphonectria hypovirus 1 is known to induce hypovirulence in *C. parasitica* by reducing pathogenic growth and sporulation, hence the virus is used in Europe for disease control. The virus has been introduced into continental Europe on multiple occasions in association with *C. parasitica* from countries such as Japan, China, and Korea, which are known to be the geographical origin of the fungus. Since those introductions, both *C. parasitica* and CHV1 have spread widely. Originally, such introductions are most likely to have occurred through the plant trade and/or the importation of Asiatic planting stock, often intended for use in breeding resistance programs to chestnut ink disease caused by *Phytophthora cinnamomi* and *P. cambivora*. Six genetically distinct CHV1 subtypes have been identified in Europe (I, D, E, F1, F2 and G) [25]. Subtype I (also known as the Italian subtype), which is the only subtype that has been detected in England [15,24], is the most widespread, because it is commonly associated with mild hypovirulence. It is dominant in Italy, Switzerland, south-eastern France, Greece [26], Bosnia [27], Croatia [28], Slovenia [29], Macedonia [30], Turkey [31], and now also England [15,24], where the haplotype exactly matches haplotype E-5. The European distribution of the E-5 haplotype has not been well studied. It was described as a rare haplotype, and it has been introduced in an experimental site near Monthey (Switzerland), where it became widely established [32].

To the best of our knowledge, this is the first time that the effect of temperature on CHV1 has been evaluated. The fact that the biological replicate effect was not significant along the different models, and the original virus concentration being significant, gave a high level of confidence to the whole assay.

The low prevalence of CHV1 (17 out of 350 isolates) and the naturally low virus concentration in these isolates (1.9 to 48.1 ng/µL of viral amplicon after RT-PCR, [15,24]) in England is most likely related to a mean monthly temperature below 7.7 °C between November and April and a mean of 15.2 °C the rest of the months, over the last 8 years [33]). In contrast, the higher viral loads in the warmer temperate areas of continental Europe [34], leads us to hypothesize that the concentration and hypovirulence of this mycovirus is proportionally related to higher mean temperatures around 25 °C [22], therefore the prevailing climate is likely to have a significant influence on its success as a biocontrol agent.

That temperature can modulate the outcome of the chestnut blight fungus and its CHV1 hypovirus interaction was already suggested by a previous study [35]. Somehow, opposite effects (mycovirus-mediated temperature tolerance) have been found for examples of the Curvularia thermal tolerance virus infecting *Curvularia protuberata* [36] and sometimes in the Heterobasidion RNA virus 6 infecting *Heterobasidion annosum sensu lato* [37].

Considering the mentioned hypothesis, we analysed the accumulation of the CHV1 virus at different temperatures and its possible effect on the growth of highly infected (gained by transmissions [22]) British isolates of *C. parasitica*. The results revealed a clear relationship between the virus concentration and temperature. The accumulation of CHV1 within all six virus-infected cultures was shown to be higher between 15 to 25 °C than at lower or higher temperatures.

On the other hand, temperature regulates the fungal growth, with 30 °C the optimum temperature for *C. parasitica* in the present study, which could explain why the chestnut blight situation in England is also quite stable, as temperatures are generally much lower than this [24]. Taken together, these results suggest that temperature modulates both the fungal growth and the virus accumulation. CHV1 infection was substantially almost neutral for the growth of *C. parasitica* at 5, 10, 15 and 30 °C but had a reducing effect at 20 and 25 °C, which makes sense with the highest detected transcription levels. The same fungal isolates proved to be less pathogenic in a previous study compared to non-infected isolates when inoculated into chestnut branches and seedlings that were constantly kept at 25 °C [22].

One conclusion noted is that the distribution of the virus load along the different parts of a fungal colony is not uniform, tending to be more concentrated in the central parts than in the periphery of the colony, as suggested here in Figure 4, but also indicated previously [38]. As another conclusion, since the highest monthly mean air temperatures in England are typically recorded in July and August of each year (since 2015 the highest monthly mean temperature was measured in July 2018, at 18.8 °C), the best season to detect operative virus in England and/or to perform inoculations with the highly infected isolates gained by transmissions will be summer. Current weather patterns and further climate change could increase the amount of time that the temperature is over 25 °C, as well as widen the geographical areas experiencing those temperatures, which may have a clear impact both on the fungus (growth promotion) and the virus (virulence increment) and thus in the outcome of this important fungal, forest pathosystem.

## Figures and Tables

**Table 1:** Virus-infected and virus-free *Cryphonectria parasitica* strains used in this study.

Treatment Number	Strain	Transmitted from	VCG (*vic* Genotype)	Mating Type	Virus Haplotype
1	FTC687	(SDA540 M2273)	EU10 (2122-11)	MAT-2	E-5
2	WAR706	(WAP125 M2273)	EU9 (2111-11)	MAT-2	E-5
3	POWP709	(WAP125 M2273)	EU9 (2111-11)	MAT-2	E-5
4	FTC687	(SDA540 M2357)	EU10 (2122-11)	MAT-2	L-18
5	WAR706	(WAP125 M2357)	EU9 (2111-11)	MAT-2	L-18
6	POWP709	(WAP125 M2357)	EU9 (2111-11)	MAT-2	L-18
7	FTC687 NON-INFECTED	Virus-free	EU10 (2122-11)	MAT-2	N/A
8	WAR706 NON-INFECTED	Virus-free	EU9 (2111-11)	MAT-2	N/A
9	POWP709 NON-INFECTED	Virus-free	EU9 (2111-11)	MAT-2	N/A

**Table 2:** Model tests of between-subjects effects with Type-III Analysis of Variance (ANOVA) for testing the effect of several parameters on virus concentration and virus copy number when using cellophane PDA plates. Shaded areas represent significant effects while non-shaded areas are non-significant.

	**Virus Concentration (ng/µL)**				**Virus Copy Number**			
	**Model**	**Temperature**	**Fungal Replicate**	**Viral Strain**	**Model**	**Temperature**	**Fungal Replicate**	**Viral Strain**
Type III Sum of Squares	6,012,136.81	5,978,713.19	13,605.38	19,818.23	685,529,198,694,467.0	604,331,700,009,547.0	2,224,111,442,904.95	78,973,387,242,015
df	8	5	2	1	8	5	2	1
Mean Square	751,517.10	1,195,742.63	6802.69	19,818.23	85,691,149,836,808.40	120,866,340,001,909.5	1,112,055,721,452.47	78,973,387,242,015
Observed Power	1.00	1.00	0.06	0.08	0.99	0.99	0.06	0.58
R^2^	0.50	0.50	0.50	0.50	0.29	0.29	0.29	0.29
F	12.66	20.15	0.11	0.33	5.19	7.32	0.06	4.78
*p*	0.0001	0.0001	0.89	0.56	0.0001	0.0001	0.93	0.03
	**Virus Concentration/Extracted RNA (ng/µL)**				**Virus Copy Number/Extracted RNA**			
	**Model**	**Temperature**	**Fungal Replicate**	**Viral Strain**	**Model**	**Temperature**	**Fungal Replicate**	**Viral Strain**
Type III Sum of Squares	699,833.66	609,462.93	86,195.18	4175.54	145,068,661,314.46	136,296,027,364.27	1,366,249,325.82	7,406,384,624.36
df	8	5	2	1	8	5	2	1
Mean Square	87,479.20	121,892.58	43,097.59	4175.54	18,133,582,664.30	27,259,205,472.85	683,124,662.91	7,406,384,624.36
Observed Power	0.93	0.94	0.30	0.06	1.00	1.00	0.09	0.43
R^2^	0.19	0.19	0.19	0.19	0.39	0.39	0.39	0.39
F	2.90	4.04	1.42	0.13	7.94	11.94	0.29	3.24
*p*	0.006	0.002	0.24	0.71	0.0001	0.0001	0.74	0.05
	**Virus Concentration/Mycelial Weight (ng/µL)**				**Virus Copy Number/Mycelial Weight**			
	**Model**	**Temperature**	**Fungal Replicate**	**Viral Strain**	**Model**	**Temperature**	**Fungal Replicate**	**Viral Strain**
Type III Sum of Squares	7,634,052,101.53	7,540,863,471.70	79,309,812.22	13,878,817.59	33,464,956,110,528,400	29,013,085,112,384,300	321,942,634,010,970.6	4,129,928,364,133,164
df	8	5	2	1	8	5	2	1
Mean Square	954,256,512.69	1,508,172,694.34	39,654,906.11	13,878,817.59	4,183,119,513,816,050.00	5,802,617,022,476,860.00	160,971,317,005,485.30	412,992,836,413,3164.00
Observed Power	1.00	1.00	0.22	0.09	0.98	0.98	0.07	0.50
R^2^	0.67	0.67	0.67	0.67	0.24	0.24	0.24	0.24
F	25.25	39.90	1.04	0.36	4.00	5.56	0.15	3.95
*p*	0.0001	0.0001	0.35	0.54	0.0001	0.0001	0.85	0.04

**Table 3:** Model tests of between-subjects effects with Type-III Analysis of Variance (ANOVA) for testing the effect of several parameters on colony diameter in general and with only positives. Shaded areas represent significant effects while non-shaded areas are non-significant.

	Colony Diameter (Overall)				Colony Diameter (without Negatives)			
	Model	Temperature	Fungal Replicate	Viral Strain	Model	Temperature	Fungal Replicate	Viral Strain
Type III Sum of Squares	177.93	1170.40	0.20	7.32	745.70	745.60	0.075	0.03
df	9	5	2	2	8	5	2	1
Mean Square	130.88	234.08	0.10	3.66	93.21	149.12	0.037	0.03
Observed Power	1.00	1.00	0.07	0.87	1.00	1.00	0.06	0.05
R^2^	0.92	0.92	0.92	0.92	0.94	0.94	0.94	0.94
F	211.43	378.14	0.16	5.91	222.94	356.66	0.08	0.07
*p*	0.0001	0.0001	0.84	0.003	0.0001	0.0001	0.91	0.78

**Table 4:** Model tests of between-subjects effects with Type-III Analysis of Variance (ANOVA) for testing the effect of several parameters on virus concentration and virus copy number when using broth cultures. Shaded areas represent significant effects while non-shaded areas are non-significant.

	Virus Concentration (ng/µL)				Virus Copy Number			
	Model	Temperature	Fungal Replicate	Viral Strain	Model	Temperature	Fungal Replicate	Viral Strain
Type III Sum of Squares	1,556,364.86	207,684.71	81643.77	1,267,036.37	14,143,441,842.83	3,703,425,992.14	1,119,836,716.47	9,320,179,134.21
df	8	5	2	1	8	5	2	1
Mean Square	194,545.60	41,536.94	40,821.88	1,267,036.37	1,767,930,230.35	740,685,198.42	559,918,358.24	9,320,179,134.21
Observed Power	1.00	0.83	0.55	1.00	1.00	0.76	0.38	1.00
R^2^	0.52	0.52	0.52	0.52	0.32	0.32	0.32	0.32
F	13.74	2.93	2.88	89.54	5.94	2.49	1.88	31.33
*p*	0.0001	0.01	0.06	0.0001	0.0001	0.03	0.15	0.0001

## Data Availability

The data that support the findings of this study are available under reasonable request.
